# Clinical outcomes of a new four-haptic hydrophobic presbyopia-correcting intraocular lens

**DOI:** 10.1038/s41598-023-35377-0

**Published:** 2023-05-24

**Authors:** Woong-Joo Whang, Tae-im Kim, Hungwon Tchah, Kyungmin Koh

**Affiliations:** 1 Department of Ophthalmology, Yeouido St. Mary’s Hospital, The Catholic University College of Medicine, Seoul, Republic of Korea; 2 Department of Ophthalmology, Severance Hospital, Institute of Vision Research, Yonsei University College of Medicine, Seoul, Republic of Korea; 3 Department of Ophthalmology, Kim’s Eye Hospital, Konyang University College of Medicine, 136 Youngshinro, Youngdeungpo-gu, Seoul, 07301 Republic of Korea

**Keywords:** Lens diseases, Refractive errors

## Abstract

A new presbyopia-correcting intraocular lens (IOL) combining bifocal and extended-depth-of-focus profiles (Symbiose: Artis Symbiose Plus; Cristalens Industrie, Lannion, France) was introduced. We compared the output with that of a standard monofocal IOL (PL E: Artis PL E). The two four-haptic hydrophobic IOLs were made of the same material from the same company. Cataract patients bilaterally implanted with either PL E or Symbiose between November 2021 and August 2022 were reviewed. The principal measures of the postoperative results were uncorrected distance visual acuity (UDVA); corrected distance VA (CDVA); uncorrected intermediate VA; uncorrected near VA; objective optical quality; and distance-corrected defocus curves. This study included forty-eight patients (96 eyes), with 22 and 26 patients (44 and 52 eyes, respectively) being implanted with PL E and Symbiose, respectively. All patients received the same type of IOL implanted in both eyes. The average age of patients was 70.9 ± 7.1 and 60.0 ± 8.5 years in PL E and Symbiose groups, respectively, with significantly younger patients in Symbiose group (*p* < 0.001). Both IOLs displayed excellent UDVA and CDVA with no statistical difference (*p* = *0.081 (monocular UDVA), p* = *0.599 (monocular CDVA), p* = *0.204 (binocular UDVA), and p* = *0.145 (binocular CDVA)*). In comparison with PL E group, Symbiose group showed significantly superior postoperative intermediate and near VA (*p* < 0.001). PL E group showed significantly superior objective optical quality compared with Symbiose group (*p* < 0.001). Symbiose provides a continuous range of vision that ensures a seamless transition from far to near with no discontinuity. It also delivers a smooth defocus curve with a larger landing area than the PL E. But the objective optical quality was better in PL E.

## Introduction

As life expectancy increases and older adults continue to participate in working life, presbyopia has developed into a common visual disability^1^. During the last decade, a considerable number of presbyopia-correcting intraocular lenses (IOLs) have been introduced. Multifocal IOLs have more than one focal point for reducing the frequency of spectacle dependence^2^. Extended-depth-of-focus (EDOF) IOLs using technology distribute light through an extended area of focus have been developed for a continuous range of vision^3^. A presbyopia-correcting IOL combining these two principal concepts was also introduced recently^4,5^.

Artis Symbiose Plus (Symbiose; Cristalens Industrie, Lannion, France) combining both bifocal and EDOF method was recently released^6^. Since the release of Symbiose, two laboratory investigation studies have been reported^6,7^. but no studies have reported on the performance of this IOL targeting patients in real clinical settings.

Consequently, we attempted to assess the performance of the IOL by comparing it with a monofocal Artis PL E (PL E; Cristalens Industrie, Lannion, France) IOL on the same platform.

## Materials and methods

### Participants

This single-center, retrospective, comparative study was conducted in accordance with the tenets of the Declaration of Helsinki and was approved by the Institutional Review Board (IRB file number: 2022-05-006) of Kim’s Eye Hospital, Seoul, Republic of Korea. The IRB granted an exemption to written informed consent because the retrospective study data were analyzed anonymously. Cataract patients bilaterally implanted with either PL E or Symbiose between November 2021 and August 2022 were examined. The study involved patients aged 40–80 years with clinically significant age-related bilateral cataracts with a follow-up period of at least 3 months. The exclusion criteria were as follow: any ocular disorders (other than cataract) that could potentially cause visual acuity (VA) loss, any anterior segment pathologies that could significantly affect outcomes, any type of corneal disorders, any eye infection, any degenerative visual disorders, zonular-break risk factors, intraoperative or postoperative complications that can affect VA, pupil anomalies of shape or dynamics, deep amblyopia, diabetic retinopathy, uncontrolled glaucoma, macular disease or retinopathy, neuro-ophthalmic diseases, choroidal hemorrhage, microphthalmia, previous intraocular surgery, previous corneal surgery, previous laser refractive surgery, and corneal astigmatism greater than 1.0 diopter (D).

VA assessment was done using the Early Treatment Diabetic Retinopathy Study (ETDRS) chart under photopic light conditions. Uncorrected distance VA (UDVA) and corrected distance VA (CDVA) were evaluated at 6 m, uncorrected intermediate VA (UIVA) was measured at 66 cm, and uncorrected near VA (UNVA) was evaluated at 40 and 33 cm. All VA and defocus curves tests using defocus lenses from + 1.50 to − 4.00 D in 0.5 D steps were carried out monocularly and binocularly at 3 months after surgery^8^.

VA values measured using the ETDRS chart were converted to logarithm of the minimum angle of resolution (LogMAR) scale for statistical analysis^9^. The Barrett Universal II formula of a high-resolution anterior segment swept-source optical coherence tomography (ANTERION, Heidelberg Engineering GmbH, Germany) was used for the calculation of IOL power and predicted postoperative refractive error (RE). The IOL power assumed for the calculations was the closest to that of emmetropia.

The RE was assessed as the difference between the postoperative spherical equivalent (SE) and the predicted SE^10^. The mean absolute error (MAE) was calculated as the average absolute value of the RE^11^. We observed the RE and MAE as measures of refractive predictability and the accuracy of the IOL power calculation.

The measurement of pupil size was conducted with ANTERION in photopic illumination.

### Intraocular lenses

**Figure 1:**
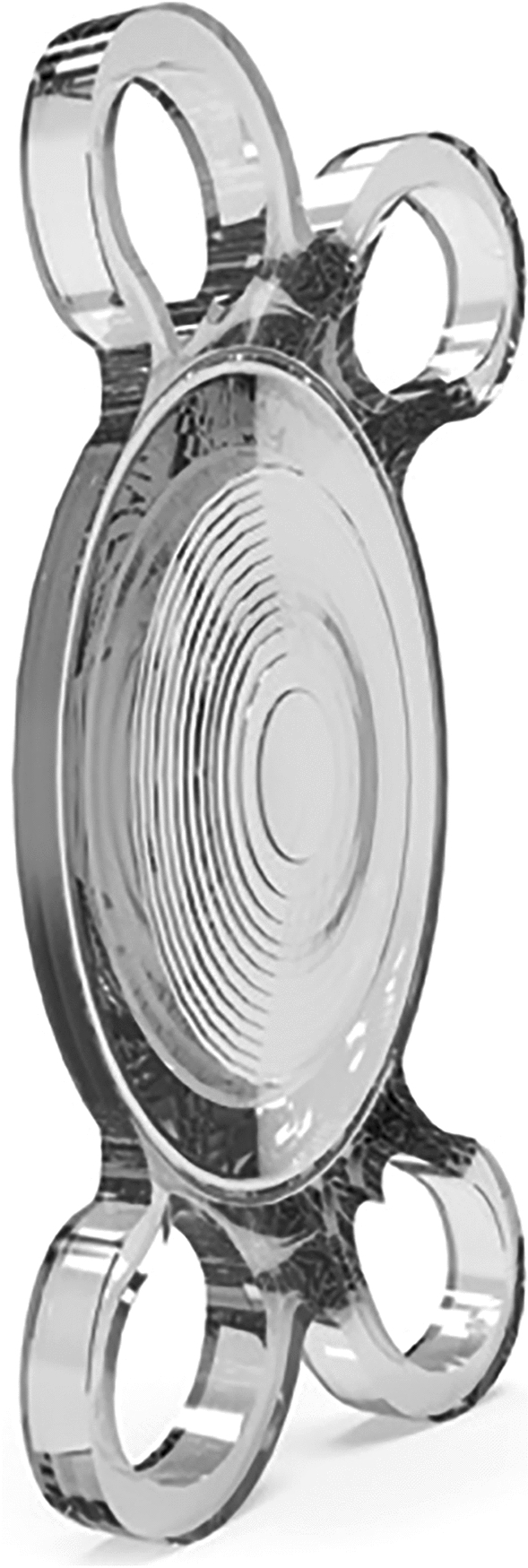
The illustration shows a four-haptic hydrophobic presbyopia-correcting intraocular lens (Artis Symbiose Plus) with 12 diffractive rings on its surface.

Symbiose IOL combining the bifocal and EDOF method was recently released and received the CE mark in 2018. Its 12 diffractive rings are designed to offer a continuous vision from intermediate to near distance while preserving highly distinguished far vision (Fig. 1)^7^. The Symbiose IOL is available worldwide in two versions, the Mid version with a superior continuum for intermediate vision and the Plus version with a superior continuum for near vision^12^. However, because only the Plus version is imported into the Republic of Korea, only the Plus version has been implemented in both eyes.

PL E is a monofocal IOL manufactured by the same company that received a CE mark in 2014. They have the same basic design (a continuous 360-degree posterior square edge and 6.0-mm optics, which are designed for negative spherical aberration of − 0.23 μm) and are made from the same material (ultraviolet light-blocking hydrophobic acrylic material, refractive index = 1.54 at 35 °C)^7^.

### Objective optical quality assessment

The HD Analyzer (Visiometrics SL., Terrassa, Spain) measurements were made at the mesopic state with a 4-mm aperture^8^. The objective scatter index (OSI), modulation transfer function (MTF), and Strehl ratio (SR) were measured^4^. The higher the OSI, the greater the level of intraocular diffusion, resulting in lower visual quality^13^. The MTF cut-off is the peak spatial frequency that the eye can identify^14^. The SR is the ratio between the maximum intensity from the point spread function (PSF) of the aberrated eye and the maximum intensity from the PSF of the unaberrated eye^15^. Higher MTF and SR values generally imply enhanced objective optic quality^16,17^.

### Surgical procedures

A single experienced surgeon (KK) performed all the surgery using a single device (Whitestar Signature phacoemulsification system; Johnson & Johnson Vision). The 2.8 mm main corneal incision was made in the steep meridian and a 5.2 mm capsulorhexis creation was made.

### Statistical analysis

A software program (IBM SPSS Statistics for Windows, version 22.0) was used to perform statistical analyses of the data. The program was created by IBM Corporation (Armonk, New York, USA). To verify normal data distributions, the Kolmogorov–Smirnov test was used. The Pearson chi-square test, the Mann–Whitney U test, and the unmatched Student t-test were used to compare data from two groups. A p value of lower than 0.05 was determined to be statistically significant.

### Ethical approval

All procedures performed in studies involving human participants were in accordance with the ethical standards of the institutional and/or national research committee and with the 1964 Helsinki declaration and its later amendments or comparable ethical standards. The study was approved by the Institutional Review Board of Kim’s Eye Hospital, Seoul, Republic of Korea (2022-05-006).

### Informed consent

Considering the retrospective nature of the study and the use of de-identified patient data, the written informed consent was waived by the Institutional Review Board of Kim’s Eye Hospital, Seoul, Republic of Korea.

### Consent to participate

Informed consent was waived due to the retrospective nature of the study. Furthermore, this study does not contain any personal information that can lead to an identification of the patient.

## Results

This study included forty-eight patients (96 eyes), with twenty-two patients (44 eyes) implanted with PL E and twenty-six patients (52 eyes) implanted with Symbiose. All patients received the same type of IOL implanted in both eyes. The average age of the patients was 70.9 ± 7.1 (range, 57–79) and 60.0 ± 8.5 (range, 41–77) years in the PL E and Symbiose groups, respectively, with significantly younger patients being observed in the Symbiose group (*p* < 0.001). The other preoperative parameters did not differ between the two groups (Table 1). A total of 45% (10/22) and 50% (13/26) of the patients were women in the PL E and Symbiose groups, respectively (*p* = 0.811). In the PL E group, the mean preoperative monocular UDVA (LogMAR) and CDVA (LogMAR) were 0.44 ± 0.35 and 0.22 ± 0.20, respectively. In the Symbiose group, they were 0.56 ± 0.40 and 0.19 ± 0.14, respectively. For preoperative VA, no statistical differences were observed between the two groups (*p* = 0.081 (monocular UDVA), *p* = 0.599 (monocular CDVA), *p* = 0.204 (binocular UDVA), and *p* = 0.145 (binocular CDVA)).

**Table 1:** Preoperative parameters for patient groups.

Parameter	PL E	Symbiose Plus	*p*-value
Patients/eyes, n	22/44	26/52	N/A
Female, n	10 (45%)	13 (52%)	0.811*
Age (years)	70.9 ± 7.1 (57 to 79)	60.0 ± 8.5 (41 to 77)	< 0.001^†^
UDVA (LogMAR)^#^	0.44 ± 0.35 (0.1 to 2.3)	0.56 ± 0.40 (0.1 to 1.7)	0.201^†^
CDVA (LogMAR)^#^	0.22 ± 0.20 (0.1 to 1.4)	0.19 ± 0.14 (0 to 0.5)	0.311^†^
Sphere (D)	− 0.08 ± 2.00 (− 3.5 to 3.0)	− 0.34 ± 2.39 (− 7.5 to 3.0)	0.877^†^
Cylinder (D)	− 0.65 ± 0.25 (− 1.0 to 0.0)	− 0.60 ± 0.10 (− 1.0 to 0.0)	0.396^†^
ACD (mm)	3.32 ± 0.37 (2.6 to 4.0)	3.36 ± 0.35 (2.4 to 4.0)	0.535^‡^
Axial length (mm)	23.85 ± 0.74 (22.2 to 26.0)	23.90 ± 1.58 (22.1 to 28.0)	0.356^†^
Pupil size (mm)	4.08 ± 0.73 (2.97 to 6.49)	4.13 ± 0.79 (2.99 to 6.34)	0.758^‡^
Flat keratometry (D)	43.81 ± 1.25 (41.3 to 45.6)	43.36 ± 1.29 (39.0 to 45.9)	0.094^†^
Steep keratometry(D)	44.51 ± 1.26 (42.1 to 46.5)	44.07 ± 1.30 (40.5 to 46.5)	0.091^‡^

N/A, not applicable; UDVA, uncorrected distance visual acuity; CDVA, corrected distance visual acuity; LogMAR, logarithm of the minimum angle of resolution; D, diopter; ACD, anterior chamber depth.

*Chi-square test.

^†^Mann‑Whitney U test.

^‡^Unpaired student t‑test.

^#^Pre-operative visual acuity was measured monocularly.

The results of the three-month post-operative VA assessment for both groups are shown in Table 2. Monocular UDVA and CDVA showed remarkable results in both groups, with no significant differences between the groups. In the PL E group, the mean postoperative monocular UDVA (LogMAR) and CDVA (LogMAR) were 0.02 ± 0.04 and 0.02 ± 0.04, respectively. In the Symbiose group, these values were 0.04 ± 0.06 and 0.02 ± 0.05, respectively. In the PL E group, the mean postoperative monocular UIVA (at 66 cm, logMAR), UNVA (at 40 cm, logMAR), and UNVA (at 33 cm, logMAR) were 0.24 ± 0.09, 0.32 ± 0.15, and 0.41 ± 0.12, respectively. These values were 0.14 ± 0.09, 0.06 ± 0.08, and 0.10 ± 0.11, respectively, in the Symbiose group. The three parameters were significantly better in the Symbiose group than in the PL E group (*p* < 0.001).

**Table 2:** Clinical results after 3 months of surgery.

Parameter	PL E	Symbiose Plus	*p*-value
Monocular visual outcome			
UDVA (LogMAR)	0.02 ± 0.04 (0 to 0.1)	0.04 ± 0.06 (0 to 0.2)	0.081*
CDVA (LogMAR)	0.02 ± 0.04 (0 to 0.1)	0.02 ± 0.05 (0 to 0.2)	0.599*
UIVA (LogMAR) 66 cm	0.24 ± 0.09 (0 to 0.5)	0.14 ± 0.09 (0 to 0.4)	< 0.001*
UNVA (LogMAR) 40 cm	0.32 ± 0.15 (0.2 to 0.6)	0.06 ± 0.08 (0 to 0.3)	< 0.001*
UNVA (LogMAR) 33 cm	0.41 ± 0.12 (0.4 to 0.8)	0.10 ± 0.11 (0 to 0.3)	< 0.001*
Binocular visual outcome			
UDVA (LogMAR)	0.02 ± 0.04 (0 to 0.1)	0.02 ± 0.06 (0 to 0.2)	0.204*
CDVA (LogMAR)	0.02 ± 0.04 (0 to 0.1)	0.01 ± 0.04 (0 to 0.2)	0.145*
UIVA (LogMAR) 66 cm	0.21 ± 0.08 (0.2 to 0.5)	0.09 ± 0.04 (0 to 0.3)	< 0.001*
UNVA (LogMAR) 40 cm	0.28 ± 0.07 (0.2 to 0.6)	0.04 ± 0.06 (0 to 0.2)	< 0.001*
UNVA (LogMAR) 33 cm	0.37 ± 0.11 (0.2 to 0.8)	0.07 ± 0.07 (0 to 0.3)	< 0.001*
Target SE (D)	− 0.02 ± 0.12 (− 0.22 to 0.30)	− 0.02 ± 0.10 (− 0.23 to 0.24)	0.786^†^
Refractive spherical (D)	0.33 ± 0.40 (− 0.50 to 1.50)	0.25 ± 0.45 (− 0.50 to 1.50)	0.309*
Refractive cylinder (D)	− 0.68 ± 0.31 (− 1.00 to 0)	− 0.55 ± 0.35 (− 1.00 to 0)	0.058*
MRSE (D)	− 0.01 ± 0.33 (− 0.75 to 1.00)	− 0.02 ± 0.38 (− 0.75 to 1.00)	0.971*
Refractive error (D)	− 0.01 ± 0.35 (− 1.02 to 0.62)	0.01 ± 0.38 (− 0.90 to 0.71)	0.962^†^
Mean absolute error (D)	0.26 ± 0.21 (0.01 to 0.87)	0.33 ± 0.20 (0.04 to 1.02)	0.056*

UDVA, uncorrected distance visual acuity; CDVA, corrected distance visual acuity; UIVA, uncorrected intermediate visual acuity; UNVA, uncorrected near visual acuity; LogMAR, logarithm of the minimum angle of resolution; SD, standard deviation; SE, spherical equivalent; MRSE, manifest refraction spherical equivalent; D, diopter; RE, refractory error; n, number.

* Mann‑Whitney U test.

^†^Unpaired student t‑test.

Binocular UDVA and CDVA showed outstanding results in both groups, with no significant differences between the groups. In the PL E group, the mean postoperative binocular UDVA (LogMAR) and CDVA (LogMAR) were 0.02 ± 0.04 and 0.02 ± 0.04, respectively. These values were 0.02 ± 0.06 and 0.01 ± 0.04, respectively, in the Symbiose group. In the PL E group, the mean postoperative binocular UIVA (at 66 cm, logMAR), UNVA (at 40 cm, logMAR), and UNVA (at 33 cm, logMAR) were 0.21 ± 0.08, 0.28 ± 0.07, and 0.37 ± 0.11, respectively. These values were 0.09 ± 0.04; 0.04 ± 0.06; and 0.07 ± 0.07, respectively, in the Symbiose group. The Symbiose group showed clearly superior results compared with the PL E group for these three parameters (*p* < 0.001).

The mean target SE was − 0.02 ± 0.12 D and − 0.02 ± 0.11 D in the PL E and Symbiose groups, respectively (*p* = 0.786). The mean refractive spherical was 0.33 ± 0.40 D in the PL E group and 0.25 ± 0.45 D in the Symbiose group (*p* = 0.309). The mean refractive cylinder was − 0.68 ± 0.31 D in the PL E group and − 0.55 ± 0.35 D in the Symbiose group (*p* = 0.058). The mean MRSE was − 0.21 ± 0.34 D in the PL E group and − 0.09 ± 0.31 D in the Symbiose group (*p* = 0.971. The mean RE was 0.04 ± 0.33 D in the PL E group and 0.03 ± 0.37 D in the Symbiose group (*p* = 0.962). The mean MAE was 0.26 ± 0.21 D in the PL E group and 0.33 ± 0.20 D in the Symbiose group (*p* = 0.056). No significant differences were observed between the two groups for these six parameters.

**Figure 2:**
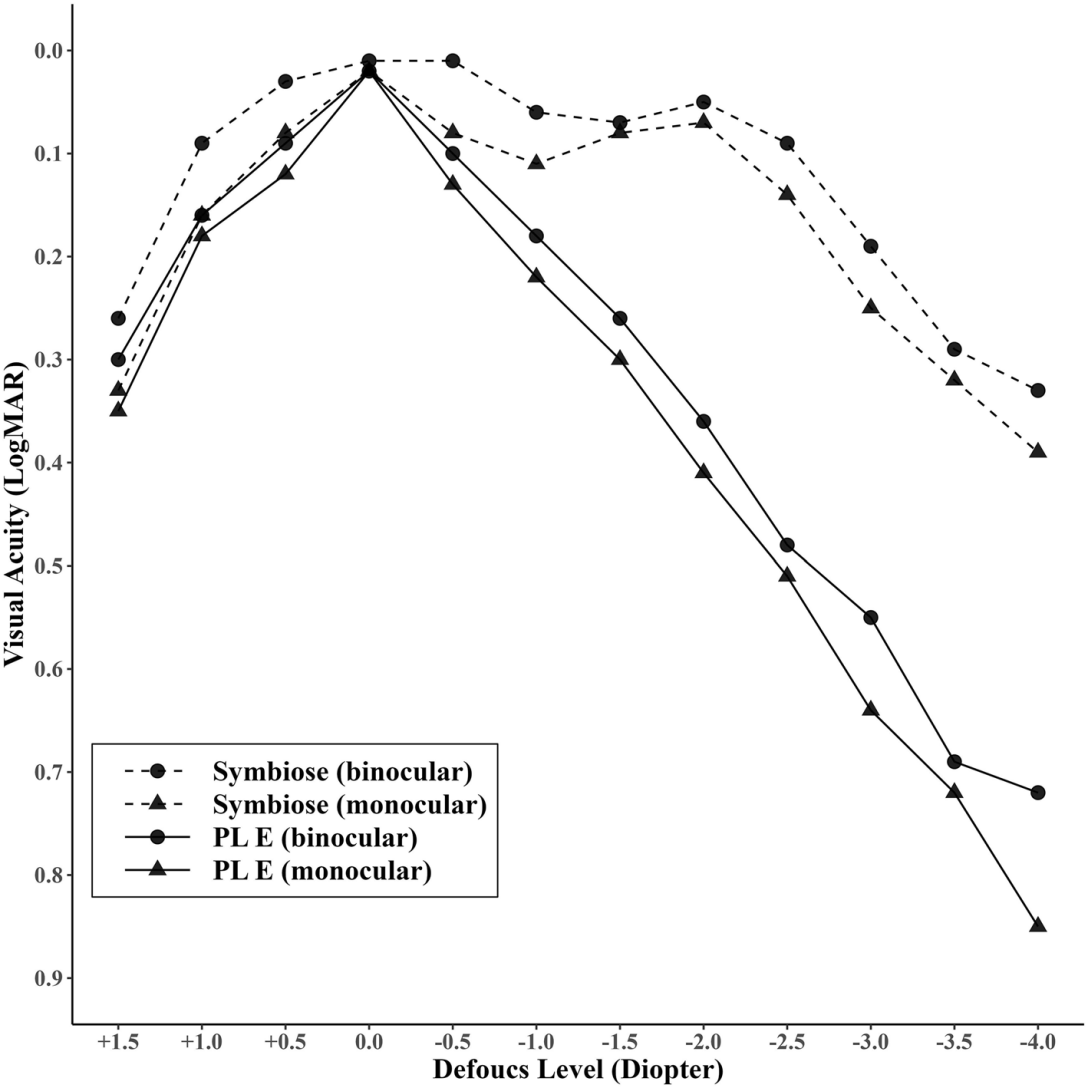
Distance-corrected defocus curves obtained from the Artis PL E and Artis Symbiose Plus groups.

Distance-corrected defocus curves were assessed 3 months after surgery at 6 m under mesopic light conditions (fixed at 10 lx), monocularly and binocularly. These defocus curves demonstrated that Symbiose presented a wider DOF range than PL E (Fig. 2). The monocular distance-corrected defocus curves of Symbiose indicated that the mean VA remains at or above 0.11 LogMAR in the range of + 0.5 to − 2.0 D of defocus. The binocular distance-corrected defocus curves of Symbiose showed that between defocus range of + 1.0 to − 2.5 D of defocus, the VA remained ≥ 0.1 logMAR. By contrast, the defocus curves for PL E showed a steep decline.

Table 3 presents the objective optical quality results of the evaluation using the HD analyzer. The OSI, MTF cutoff, and SR of PL E were 2.18 ± 1.32, 28.28 ± 13.01, and 0.17 ± 0.08, respectively, while those of Symbiose were 4.63 ± 4.42, 16.21 ± 11.16, and 0.10 ± 0.05, respectively. Values for all these parameters were superior in PL E compared to Symbiose (*p* < 0.001), indicating that PL E has a better objective optical quality than Symbiose.

**Table 3:** Optical quality parameters evaluated by the HD analyzer after 3 months of surgery.

Parameter	PL E	Symbiose Plus	*p*-value
OSI	2.18 ± 1.32 (0.6 to 6.6)	4.63 ± 4.42 (0.8 to 18.7)	< 0.001*
MTF cutoff (c/deg)	28.28 ± 13.01 (17.22 to 33.15)	16.21 ± 11.16 (2.6 to 50.7)	< 0.001*
Strehl ratio	0.17 ± 0.08 (0.07 to 0.38)	0.10 ± 0.05 (0.04 to 0.24)	< 0.001*

OSI, objective scatter index; MTF, modulation transfer function; SD, standard deviation.

*Mann‑Whitney U test.

## Discussion

Different types of IOLs are used in cataract surgery, specifically multifocal and EDOF IOLs^18^. Multifocal IOLs have multiple optical zones with different refractive powers that allow for good VA at different distances, such as near, intermediate, and far. However, they may cause a reduction in contrast sensitivity and an increase in glare, which can affect the quality of vision^19,20^. In contrast, EDOF IOLs are designed to enhance visual range, especially at intermediate distances while maintaining a high level of continuous VA^21^.

The defocus curve is a graphical representation of a patient's VA at various levels of defocus^22^. It is commonly used to assess the DOF for presbyopia-correction. It serves as a visual accomplishment marker, allowing doctors to evaluate the performance of the IOL and determine if it provides the desired level of VA at different distances^23–25^. Symbiose provided a smooth defocus curve with a broader landing area than the PL E (Fig. 2). Symbiose is known to offer a clear vision range from + 1.50 to − 3.75 D of defocus uninterruptedly while maintaining high contrast vision^7^. In this study, Symbiose maintained binocular VA better than or equal to 0.3 LogMAR in the + 1.5 D to − 3.5 D interval. However, PL E kept binocular VA above or equal to 0.3 LogMAR only in the short interval of + 1.5D to − 1.5D (Fig. 2). In one study, it was shown that there is a secondary peak in the VA at − 2.5 D of defocus^6^. Likewise, in our study, the peak of secondary VA was observed at − 2.0 D of defocus.

The HD Analyzer is a tool that can be used to evaluate the optical quality of IOLs and has been found to have good repeatability^26^. Studies have shown that the objective OSI value obtained from the HD analyzer is strongly associated with subjective levels of glare^4,27^. Not surprisingly, the objective optical quality of PL E, a standard monofocal IOL, was superior to Symbiose. This is a common finding in studies comparing monofocal IOL with presbyopia-correcting IOL^28,29^.

Similar to Symbiose, Tecnis Synergy (Johnson & Johnson Vision, Santa Ana, CA, USA) is a bifocal IOL combined with EDOF technology for a greater range of vision^29^. A comparison of the performance and characteristics of these two IOLs under the same conditions would be meaningful. Comparing the performance and characteristics of these two IOLs under the same conditions would provide useful information for understanding the differences between these two IOLs and how they may perform in different situations. A study that compared these two IOLs using the same measurement methods, such as VA, contrast sensitivity, glare, patient satisfaction, and quality of vision, would provide a more accurate and meaningful comparison of their performances.

This study has some limitations, including its retrospective design and non-randomization, which may affect the interpretation of the results. Additionally, the evaluation of objective optical quality with diffractive IOLs can be limited due to the light loss caused by the diffraction patterns^30^. The HD analyzer can account for this diffusion by measuring higher OSI values in the diffraction rings^29^. The study also found that the individuals in the Symbiose group were younger, which may have influenced the results due to the effect of age on pupil size. Pupil size may decline with age, which can influence the DOF and near vision^31^. In Republic of Korea, you only need to pay $160 for monocular surgery if you are operating on monofocal IOL. But if you choose a presbyopia-correcting IOL, you have to pay fifteen times more. For this reason, the elderly, who are economically vulnerable and have a relatively weak desire for presbyopia correction, are less likely to choose a presbyopia-correcting IOL. Future studies will be required to compare results by removing the age gap between the two groups. Finally, there were no subjective evaluations of the quality of vision, such as contrast sensitivity tests, which would have provided additional information regarding the properties of each IOL.

In summary, Symbiose provides a continuous range of vision that ensures a seamless transition from far to near with no discontinuity. It also delivers a smooth defocus curve with a larger landing area than the PL E. But the objective optical quality was better in PL E.

## Supplementary Information

Supplementary Information.

## Data Availability

The datasets generated and/or analyzed during this study are available in a supplementary Excel file attached to the submission.
